# Development and Psychometric Evaluation of Family Caregivers’ Hardiness Scale: A Sequential-Exploratory Mixed-Method Study

**DOI:** 10.3389/fpsyg.2022.807049

**Published:** 2022-04-01

**Authors:** Lida Hosseini, Hamid Sharif Nia, Mansoureh Ashghali Farahani

**Affiliations:** ^1^School of Nursing & Midwifery, Iran University of Medical Sciences, Tehran, Iran; ^2^School of Nursing and Midwifery, Mazandaran University of Medical Sciences, Sari, Iran

**Keywords:** family caregivers, Alzheimer, hardiness, validity, psychometric, scale

## Abstract

**Objective:**

Caring for patients with Alzheimer’s disease (AD) is a stressful situation and an overwhelming task for family caregivers. Therefore, these caregivers need to have their hardiness empowered to provide proper and appropriate care to these older adults. From the introduction of the concept of hardiness, few studies have been conducted to assess the hardiness of caregivers of patients with AD. Presumably, one reason for this knowledge gap is the lack of a proper scale to evaluate hardiness in this group. This study was conducted to develop a reliable and valid Family Caregivers’ Hardiness Scale (FCHS) to measure this concept accurately among Iranian family caregivers sample.

**Methods:**

This study is a cross-sectional study with a sequential-exploratory mixed-method approach. The concept of family caregivers’ hardiness was clarified using deductive content analysis, and item pools were generated. In the psychometric step, the samples were 435 family caregivers with a mean age of 50.26 (SD ± 13.24), and the data were gathered *via* an online form questionnaire. In this step, the items of the FCHS were evaluated using face and content validity. Then, the factor structure was determined and confirmed using exploratory factor analysis (EFA) and confirmatory factor analysis (CFA) followed by convergent and divergent validity, respectively. Finally, scale reliability, including stability, and internal consistency were evaluated.

**Results:**

The finding revealed that FCHS consists of five factors, namely, “Religious Coping” (5 items), “Self-Management” (6 items), “Empathic Communication” (3 items), “Family Affective Commitment” (3 items), and “Purposeful Interaction” (4 items) that explained 58.72% of the total variance. The results of CFA showed a good model fit. Reliability showed acceptable internal consistency and stability.

**Conclusion:**

Based on the results of the psychometric evaluation of the FCHS, turned out that the concept of hardiness in Iranian family caregivers is a multidimensional concept that is most focused on individual-cultural values, emotional family relationships, and social relationships. The designed scale also has acceptable validity and reliability features that can be used in future studies to measure this concept in family caregivers.

## Introduction

Aging has become one of the greatest concerns around the world due to increasing life expectancy and decreasing mortality (Santos da Silva et al., 2018). Based on the WHO reports, in 2019, 703 million people aged 65 years and older worldwide, and it will reach 1.5 billion people by 2030. This increase in developing countries such as Iran will occur faster than in developed countries (World Health Organization [WHO], 2020).

Aging is a natural and inevitable process of life and is associated with a series of physical, cognitive, and emotional changes (Pashaki et al., 2015). Alzheimer’s disease (AD) is one of the most common types of cognitive disorder that affects the memory, thinking, and behavior of older adults and reduces the person’s ability to live independently (Santos da Silva et al., 2018). The Alzheimer’s Disease International (ADI) Federation estimates 35.6 million people live with AD worldwide, and it will double every 5 years after the age of 65 years (Alzheimer’s Disease International [ADI], 2017; Trevisan et al., 2019). Since older adults with AD are limited in performing their activities of daily living, they need to be supported by a formal or informal caregiver (Santos da Silva et al., 2018). Due to the interdependence between family members, declining household incomes especially in developing countries such as Iran, the lack of formal support systems, more than 81% of these patients are in need of care by family caregivers (Sharifi et al., 2016). Family caregivers are considered informal caregivers and lack training; these individuals do not receive any reimbursement for their services (Lynch et al., 2018).

The caregiver burden for family caregivers of patients with AD is heavy work, and caring for patients with AD is stressful and can become overwhelming for family caregivers. As the severity of the disease increases, it affects all aspects of these caregivers’ lives and can produce many acute and chronic physical and emotional problems for family caregivers (Armstrong et al., 2019). Thus, caregivers can be considered “invisible secondary patients” (Ashrafizadeh et al., 2021). Previous studies have shown that depression, anxiety, stress, and burnout are the most common sequela of caring for family caregivers of patients with AD. So that, more than 80% of caregivers suffer from stress and burnout, 30–40% suffer from depression, and 44% suffer from anxiety (Baharudin et al., 2019; Fujihara et al., 2019). Therefore, for these caregivers to be able to adapt properly to the situation and not suffer from the negative side effects, they need the ability, competence, and skills to adapt to the situation (Lynch et al., 2018). According to Hooker et al., personal characteristics such as hardiness can be a major factor in changing the care experience when caring for patients with AD and increase the caregivers’ ability for positive coping with these stresses (Hooker et al., 1998).

### Background

Hardiness was first proposed by Kobasa (1979). It is one of these effective personal characteristics which makes sense in the face of stressful situations and is considered as a moderating variable in the relationship between stress and its physical and psychological effects (Abdollahi et al., 2018). According to Kobasa, hardiness is a combination of attitudes and beliefs that motivate a person to do hard and strategic work in the face of stressful and difficult situations and can turn adversity into an opportunity for growth (Maddi, 2002). Accordingly, this concept consists of three components, namely, commitment, control, and challenge (Kobasa, 1979). Commitment refers to a tendency to engage in life’s activities and to have a real interest and curiosity about the world around them. Control refers to the belief that individuals can influence the events of their lives; and finally, challenge points to the belief that change, rather than stability, is a natural part of life, which creates opportunities for personal growth rather than threatening security (Maddi, 2002). Studies show the positive effect of hardiness on health and performance in different groups such as college students, cadets, nursing students, and managers in different stressful situations (Kelly et al., 2014; Abdollahi et al., 2018; Tho, 2019). One meta-analytic review showed that hardy individuals are likely to have more life satisfaction, a better job or school performance, more optimism, greater self-esteem, and a sense of coherence as well as higher mental health; but individuals with low hardiness experience more negative effects from stressful situations such as depression and anxiety (Eschleman et al., 2010).

Since caring for patients with AD is a unique and stressful situation for family caregivers, to provide proper and appropriate care to these patients, these caregivers need to have the hardiness trait in order to be empowered. As a moderating factor, hardiness can prevent problems for the caregivers such as fatigue, burnout, depression, sleep disorders, and reduced quality of life. Hardiness can also prevent the patients from neglect, abuse, poor quality care, ignoring vital needs, and aggravation of the disease (Clark, 2002; DiBartolo and Soeken, 2003). It is noteworthy that since the introduction of the concept of hardiness, few studies have assessed the hardiness of caregivers of patients with AD. Presumably, one reason for this knowledge gap is the lack of a proper scale to evaluate hardiness in this group. Several questionnaires have been developed for measuring hardiness in different groups such as students (Benishek and Lopez, 2001), bereaved parents (Lang et al., 2003), and employees (Moreno-Jiménez et al., 2014). However, the caregiving for patients with AD is completely different from the previous studies about the role of hardiness.

Therefore, considering that the Iran population is aging, AD is an age-related phenomenon, and that patients with AD are mostly cared for by family caregivers, therefore, Iran will need to prepare hardy family caregivers. Furthermore, since hardiness can be taught to individuals, nurses and therapists will be able to design appropriate interventions to improve their hardiness and thus improve care and reduce complications. Knowing the level of the caregiver’s hardiness or evaluating the effectiveness of interventions requires an accurate scale. Thus, this study was conducted to clarify the concept of hardiness in family caregivers of patients with AD and then develop a reliable and valid scale to measure this concept accurately.

## Materials and Methods

### Design

This is a cross-sectional study to evaluate the psychometrics of the Family Caregivers’ Hardiness Scale (FCHS) from July 2020 to October 2021 in family caregivers of patients with AD. It was performed in two stages: (1) qualitative by directed content analysis approach to generate items and (2) quantitative approach to assess the psychometric properties of the developed scale.

### Qualitative Study and Item Generation

The purpose of this stage was to clarify the family caregivers’ hardiness concept and make an item pool for designing the target scale. For this purpose, based on the Kobasa’s model of hardiness, the deductive directed content analysis by Elo and Kyngäs (2008) was used to clarify the concept of the family caregivers’ hardiness in caring for patients with AD. The related structures were identified, and the items were produced in two steps: reviewing the literature and examining the experiences and perceptions of the participants through interviews. The deductive-directed content analysis includes three phases, namely, preparation, organization, and reporting.

#### First Step: A Review of the Literature

Electronic databases such as PubMed, Scopus, ISI Web of Science, and Persian databases such as Magiran, SID, and Iran Medex were searched using the keywords “hardiness,” “personality hardiness,” “hardy personality,” “caregiver hardiness,” “caregivers,” “family caregivers,” “non-professional caregivers,” “spouse caregivers,” “dementia,” and “Alzheimer” with no time limit. Studies with the following inclusion criteria were selected: relevance of the study, access to the full text of the article, and English and Persian language. In this search, duplicate and irrelevant articles, studies published in non-Persian and non-English languages, and short articles such as the editorial and commentarial materials were excluded. In the initial search, a total of 3,560 English articles and 430 Persian articles were obtained. After applying the inclusion and exclusion criteria, 23 articles were entered the analysis stage to extract initial codes. In the preparation phase, the text of each article was read several times by the researcher (L.H) as a unit of analysis to immerse in the data and to provide key points and clear descriptions of each aspect of the hardiness concepts based on the Kobasa hardiness model. Then, in the organizing phase, the researchers formed an unconstrained matrix derived from the Kobasa Hardiness Model. Initial codes (*n* = 198) were classified as categories derived from the dimension of hardiness (i.e., main categories of commitment, control, and challenge and two new main categories: connection and culture). The choice of these names for the main categories was based on the hardiness concept.

#### Second Step: An Interview With Participants

##### Participants

To deeply understand the family caregivers’ hardiness concept, 14 family caregivers with a mean age of 54.57 years were selected through purposeful sampling with maximum variety and also snowball sampling from November 2020 to February 2021. Personal characteristics were as follows: nine daughters, two sons, and three spouses. Ten participants were married, three were unmarried, and one of them was a widow. Eight participants had an academic education, and six had diploma.

##### Procedure

In-depth and semi-structured interviews (30–90 min) were conducted with each participant using a combination of model-derived questions and open-ended questions. Immediately after the end of each interview, the recorded material was transcribed word by word. In the preparation phase, the researcher (L.H) listened to the recorded statements and read the written interview several times to gain an in-depth understanding of the participants’ feelings and experiences and then analyzed it using MAXQDA software version 10. In the organizing phase, similar to the review of the literature step, the researchers formed an unconstrained matrix derived from the Kobasa hardiness model, and a total of 1,604 initial codes were extracted, leaving 606 initial codes after deleting duplicates and overlapping cases. These were classified as categories derived from the dimension of hardiness (i.e., main categories of commitment, control, and challenge and two new main categories: connection and culture). Finally, in reporting phase, the results of both steps were combined (Hosseini et al., 2021). Also, all stages of directed content analysis and the findings obtained in this study were reported. The quality of findings was assessed by Lincoln and Goba’s criteria such as credibility, dependability, confirmability, and transferability (Lincoln and Guba, 1986). Finally, based on the result of the concept analysis and the extracted codes, an item pool (656) was developed. Later, during frequent meetings of the research team, writing and grammar and also overlapping and similarity of items were checked, and some items were merged or deleted. Thus, the total number of items was reduced from 656 to 97 and then to 54 items. Therefore, at this stage, the 54-item FCHS was developed to be evaluated for psychometric properties.

### Quantitative Study and Item Reduction

During this stage, face, content, and construct validity, as well as reliability, were used to evaluate the psychometric properties of the FCHS using a five-point Likert response scale, i.e., 5 (always) to 1 (never). The sample size of each stage was different, and it was explained separately in each stage.

#### Face Validity

Face validity was evaluated with qualitative and quantitative approaches. In the qualitative approach, the scale was sent to 11 family caregivers who were asked to assess the scale in terms of difficulty, relevancy, and ambiguity. All items were understandable to the participants. In the quantitative approach, we asked the same 11 family caregivers to assess the items in terms of suitability using a five-point Likert scale (5 = it is completely suitable, 4 = it is suitable, 3 = it is almost suitable, 2 = it is less suitable, and 1 = it is not suitable at all). The impact score was calculated with the formula as follows: impact score = frequency (%) × suitability. A score of >1.5 was considered acceptable (Ebadi et al., 2020).

#### Content Validity

The content validity of the FCHS was evaluated by the qualitative and quantitative approaches. In the qualitative approach, the scale was sent to 12 experts in nursing, psychology and the development of the instrument to evaluate the items in terms of grammar and wording, item allocation, and scaling. During this process, some items were modified by their feedback. In the quantitative approach, the content validity of the scale was evaluated by content validity ratio (CVR) and modified kappa coefficient (K) to ensure that the scale measures the construct of interest. In CVR, 12 experts evaluated the essentiality of FCHS in a three-point Likert scale (1 = not essential, 2 = useful but not essential, and 3 = essential). The CVR was accounted by the formula as follows: [ne – (N/2)]/(N/2), where “ne” is the number of experts who rate the items as “Essential” and *N* is the total number of experts. The result was interpreted using the Lawshe rule. The minimum acceptable CVR score was 0.56 (Lawshe, 1975). To assess K to the elimination of chance effect for each item, 11 experts evaluated the 38-item scale in terms of relevancy by the dichotomous response: (4 = relevant, 1 = irrelevant). An excellent value of kappa was considered as >0.75 (Ebadi et al., 2020).

#### Item Analysis

Before examining the construct validity, an item analysis was conducted to identify possible problems of items by computing the corrected item-total correlation. In this step, 32 family caregivers with a mean age of 52.02 ± 13.91 years were selected using convenience sampling. They completed the online form of FCHS. We considered the correlation coefficient between items lower than 0.32 or above 0.9 as criteria for removing items (Ebadi et al., 2020).

#### Construct Validity

##### Participations and Samples

The sample consisted of Iranian family caregivers of patients with AD. The inclusion criteria to participate in this study were as follows: be the family member, relatives, and friends of the patient (informal caregivers) and providing care for the patient, agreed to participate in this study, and able to use social networks such as Telegram and WhatsApp. Based on the Rule of Thumb that considers 200 participants as the adequate sample size (MacCallum et al., 1999), 435 family caregivers were recruited into this phase for two steps: 210 for evaluating exploratory factor analysis (EFA) and 225 for evaluating confirmatory factor analysis (CFA). The participants were selected using convenience sampling through social groups related to the family caregivers of patients with AD and through the introduction of people. During this phase, data were gathered online. For this purpose, the online questionnaire was created *via* Google Form, and its URL link was sent by email or social networking applications such as Telegram channel or WhatsApp for participants.

##### Measures

**FIGURE 1:**
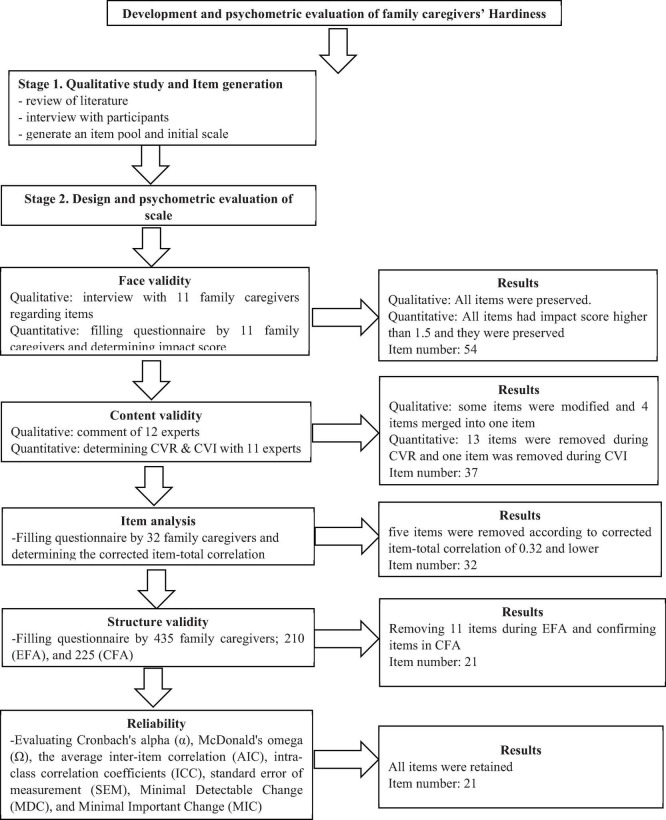
Production phases of family caregiver hardiness scale.

The questionnaire used in this step included two sections. The first section was related to the demographic characteristics such as age, gender, marital status, education level, employment status, lifestyle, relationship with the patient, average hours of care per day (h), and duration of the disease (year). The second section was FCHS with 32 items to the measuring of the family caregiver’s hardiness concept with a five-point Likert scale response (1 = never to 5 = always). The details of the production phases of FCHS are shown in Figure 1.

The construct validity of this scale was evaluated by EFA and CFA. The EFA was assessed through the maximum-likelihood method with Promax Rotation using SPSS/AMOS_26_. Furthermore, the Kaiser-Meyer-Olkin (KMO) and Bartlett’s tests were used to estimate sample adequacy and suitability. KMO values higher than 0.9 were interpreted as excellent (Pahlevan and Sharif, 2021). Horn’s parallel analysis and exploratory graph analysis were used for extracting factor structure using SPSS R-Menu_2.0_. Horn’s parallel analysis method is found to have consistent results to determine the accurate number of factors and the original scale. Horn’s parallel analysis creates eigenvalues that take into account the sampling error inherent in the dataset by creating a random score matrix of exactly the same rank and type of the variables we have in our dataset. The actual matrix values are then compared with the randomly generated matrix. The numbers of components, after successive iterations, that account for more variance than the components derived from the random data are taken as the correct number of factors to extract (Pahlevan and Sharif, 2021). Factor loading of almost 0.3 was considered to determine the presence of an item in a latent factor, and items with communalities < 0.2 were excluded from EFA. Factor loading was estimated using the following formula: CV = 5.152 ÷√ (*n* – 2), where CV is the number of extractable factors, and *N* is the sample size (Pahlevan and Sharif, 2021). Then, the factor structure determined by EFA was assessed by CFA. The CFA was performed using the maximum-likelihood method and the most common goodness-of-fit indices such as chi-square (χ^2^) test, chi-square/degree-of-freedom ratio (χ^2^/df) < 3, Comparative Fit Index (CFI) > 0.90, Incremental Fit Index (IFI) > 0.90, Tucker-Lewis Index (TLI) > 0.90, Parsimonious Normed Fit Index (PNFI) > 0.50, Parsimonious Comparative Fit Index (PCFI) > 0.50, and root mean square error of approximation (RMSEA) < 0.08 using SPSS/AMOS_26_ (Hu and Bentler, 1999; Pahlevan and Sharif, 2021).

#### Convergent and Discriminant Validity

The convergent and discriminant validity of the extracted factors was evaluated using Fornell-Larcker criteria using JASP_15.0.0_ as follows: (a) average variance extracted (AVE), (b) maximum shared squared variance (MSV), and (c) composite reliability (CR). The AVE > 0.5 and (b) CR greater than AVE was considered as the minimum requirements of convergent validity. Also, MSV less than AVE for each construct was considered the minimum requirement of the discriminant validity (Pahlevan and Sharif, 2021). In this study, the discriminant validity was assessed by a new approach developed by Heseler as Heterotrait-Monotrait Ratio (HTMT) matrix in which, to achieve discriminant validity, all values in the HTMT matrix should be less than 0.85 (Henseler et al., 2015).

#### Reliability

Reliability was evaluated using internal consistency, stability, and absolute reliability approaches using JASP_15.0.0_. The internal consistency was evaluated using Cronbach’s alpha (α), McDonald’s omega (Ω), and the average inter-item correlation (AIC). Coefficient’s α and Ω values were > 0.7, and the AIC of 0.2–0.4 was considered as an acceptable internal consistency (Sharif Nia et al., 2021). Also, CR and maximum reliability (Max H reliability) > 0.7 were used to evaluate the reliability of the construct in the structural education model (Sharif Nia et al., 2019).

The stability was evaluated by counting the intraclass correlation coefficients (ICC) of the FCHS with a two-way random effects model. For this purpose, we used the test-retest method with a 2-week interval in 15 family caregivers. The ICC value > 0.8 is considered an acceptable value of stability (Pahlevan and Sharif, 2021).

Furthermore, the absolute reliability was evaluated using standard error of measurement (SEM) by the following formula: (SEM = SD_Pooled_ × √1 − ICC) (Pahlevan and Sharif, 2021).

Finally, the responsiveness was assessed using the minimal detectable change (MDC) by using the following formula: MDC95% = SEM × √2 × 1.96 and the minimal important change (MIC) by using the following formula: MIC = 0.5 × SD of the Δ score. To interpret the MIC, it is necessary to calculate the limit of agreement (LOA). The LOA was calculated based on the following formula: LOA = *d* ± 1.96 × SD difference. If the MIC is smaller than the MDC or the MIC is not within LOA, the scale is responsive. Also, interpretability was assessed by evaluating ceiling and floor effect and MDC (Ebadi et al., 2020).

#### Multivariate Normality and Outliers

The normal distribution of data was evaluated in two ways, namely, univariate and multivariate distributions. Univariate normal distribution was evaluated using skewness (±3) and kurtosis (±7), and multivariate normality distribution was assessed by Mardia’s coefficient > 8. The data were evaluated for the outlier in two ways, namely, univariate and multivariate outliers. The univariate outlier was assessed through distribution charts, and the multivariate outlier was assessed through Mahalanobis distance *p* < 0.001 (Pahlevan and Sharif, 2021).

#### Ethical Consideration

The Iran University of Medical Sciences Research Ethics Committee approved this study (IR. IUMS. REC.1398.1229). In the beginning of each interview, the purpose of the interview was explained to the participants, and they were asked to provide written permission and informed consent to audio record their answers to questions. In addition, they were reassured that participation in the study was voluntary. Participants were assured that their information was confidential.

## Results

### Item Generation

The results of the review of literature and interview with participants were combined. Based on the results of this phase, the concept of family caregivers’ hardiness of patients with AD had five dimensions, namely, commitment, control, challenge, connection, and culture. The item pool with 656 items was generated using initial codes. Out of which 54 items were selected as items of the FCHS.

### Item Reduction

In the face validity step, the score of all items was above 1.5, and they were found to be suitable. During the assessment of content validity, in the qualitative approach, four items merged into one item according to expert panel suggestion. In quantitative approaches, the CVR of 13 items were < 0.56, and they were removed, and according to the results of kappa value, the kappa value of one item was < 0.75, and it was removed (4 items from the first dimension, 10 items from the second dimension, 1 item from the fourth dimension, and 2 items from the fifth dimension). Therefore, 17 items were removed, and the total number of the FCHS was reduced from 54 to 37 items. During the item analysis step, five items (i.e., items 12, 16, 19, 27, and 33) were also removed, because they were corrected, the item-total correlation of 0.32 and lower and the final FCHS with 32 items were entered into the factor analysis step.

#### Sociodemographic Profile of Participants

In total, 435 family caregivers with a mean age of 50.26 years (SD = 13.24) participated in this study. The number of women (50.6%) and men (49.4%) were almost equal. Most of them were married (68.7%) and daughters of patients (52.9%). The details of the sociodemographic profile of participants were shown in Table 1.

**TABLE 1:** Demographic characteristics of participants (*n* = 435).

Variables		*N* (%)
Age		50.26 ± 13.24
Gender	Female	220 (50.6)
	Male	215 (49.4)
Marital status	Single	92 (21.1)
	Married	299 (68.7)
	Divorced	14 (3.2)
	Widow	30 (6.9)
Education level	Illiterate	11 (2.5)
	Less than diploma	30 (6.9)
	Diploma	200 (46)
	Academic	194 (44.6)
Employment	Unemployed	42 (9.7)
	Employed	161 (37)
	Housewife	146 (33.6)
	Retiered	24 (5.5)
	Free	62 (14.3)
Lifestyle	Independent	262 (60.2)
	With patients	173 (39.8)
Relationship with the patient	Daughter	230 (52.9)
	Son	57 (13.1)
	Wife/midwife	57 (13.1)
	Friend	34 (7.8)
	Relative	57 (13.1)
Average hours of care per day (hour)		7.51 ± 5.51
Duration of the disease (year)		4.65 ± 2.52

**FIGURE 2:**
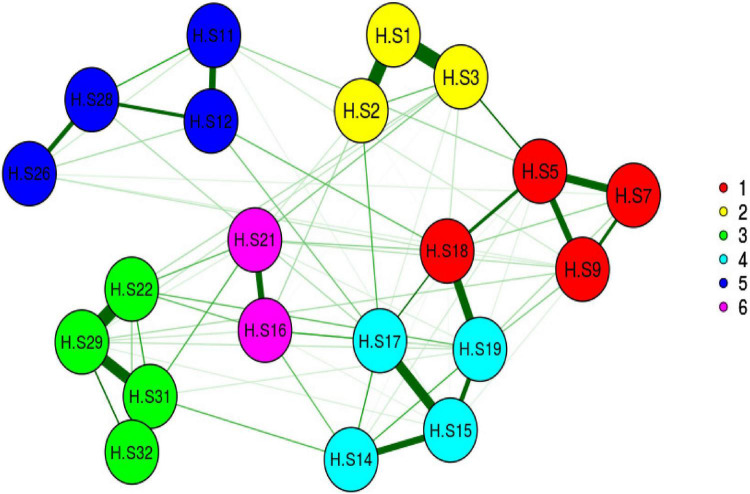
Exploratory graph analysis.

**FIGURE 3:**
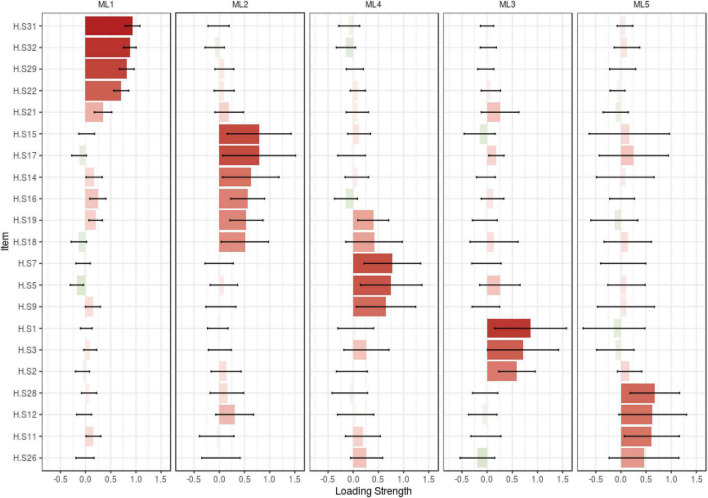
Loading strength of items in factors.

In the construct validity step, based on the results of KMO (0.935) and Bartlett’s value 2132.372 (*p* < 0.001), the sample was adequate and suitable. In this step, 11 items (items 4, 6, 8, 10, 13, 20, 23, 24, 25, 27, and 30) that were removed as the communality values of them were less than 0.2, and the factor loadings were less than 0.3, and after Promax Rotation, five-factors (21 items totally) such as “Religious Coping” (5 items), “Self-Management” (6 items), “Empathic Communication” (3 items), “Family Affective Commitment” (3 items), and “Purposeful Interaction” (4 items) were extracted. These factors explained, respectively, 16.37, 15.83, 8.96, 8.51, 9.11, and 58.72% of the total variance of family caregivers’ hardiness. The details of factor analysis results are shown in Table 2 and Figures 2, 3.

**TABLE 2:** The result of EFA on the five factors of FCHS (*N* = 210).

Factors	Q_*n*_. Item	Factor loading	h^2*^	M (SD)	Skew (kurtosis)	λ	%Variance
Religious coping	**31.** Believing in God’s help in trouble will make me stronger in the face of adversity.	0.938	0.811	4.06 (1.16)	−1.2 (0.70)	3.43	16.37
	**32.** Prayer and communion with God make me hardy against the pressure of care.	0.898	0.653	3.96 (1.33)	−1.0 (−0.27)		
	**29**. The spiritual value of patient care makes it easier for me to endure care problems.	0.890	0.776	3.68 (1.37)	−0.8 (−0.27)		
	**22.** Caring for the patient as a spiritual opportunity strengthens me.	0.776	0.674	3.59 (1.34)	−0.9 (0.03)		
	**21.** I see caring for the patient as an opportunity to repay efforts and pay my homage to the patient.	0.598	0.420	4.09 (1.14)	−1.5 (1.06)		
Self- management	**15.** By changing my mind in difficult times, I try to bear the pressure of care.	0.878	0.673	3.53 (1.07)	−0.4 (−0.47)	3.32	15.83
	**17.** With care management, I endure problems.	0.856	0.714	3.90 (0.89)	−0.8 (0.35)		
	**14.** Recalling my own abilities, I try to bear the pressure of care.	0.798	0.571	3.71 (0.88)	−0.5 (−0.58)		
	**16.** I constantly remind myself that enduring the hardships of caring is part of my job.	0.695	0.492	3.75 (1.31)	−1.0 (0.61)		
	**19.** Positive thinking helps me not to give in to difficult situations.	0.598	0.709	3.56 (1.41)	−0.7 (0.14)		
	**18.** Patience in the face of problems for my patient makes the situation bearable.	0.588	0.582	3.75 (0.84)	−0.7 (−0.16)		
Empathic communication	**7.** Understanding the involuntary nature of the patient’s problems makes it easier to endure hardships.	0.896	0.494	3.68 (1.06)	−1.0 (1.11)	1.88	8.96
	**5.** Accepting the patient’s condition makes the difficulty of caring tolerable for me	0.778	0.646	4.28 (0.28)	−0.9 (0.70)		
	**9.** Creating a sense of satisfaction in patient, makes the care easier for me.	0.689	0.502	3.96 (1.17)	−1.0 (0.39)		
Family affective commitment	**1.** My interest in my family causes me; To endure the hardships of care.	0.850	0.605	4.56 (0.71)	−1.4 (1.60)	1.78	8.51
	**3.** Love for my patient makes me endure the hardships of caring.	0.762	0.616	4.40 (0.83)	−1.1 (0.61)		
	**2.** I am responsible to my family.	0.697	0.413	4.68 (0.64)	−1.8 (1.31)		
Purposeful interaction	**28.** Talking to a doctor or nurse about a patient’s problems makes it easier for me to bear the pressure of care.	0.787	0.422	3.68 (1.02)	−0.4 (0.68)	1.91	9.11
	**11.** Gaining information about the disease through different methods (cyberspace, books, brochures, and treatment team) increases my ability to care.	0.692	0.457	3.68 (0.99)	−0.4 (0.77)		
	**12.** Sharing and exchanging ideas with family members makes it easier for me to endure problems.	0.685	0.472	3.87 (0.79)	−0.6 (−0.23)		
	**26.** Associating with friends and acquaintances makes the burden of care bearable for me.	0.589	0.244	3.81 (1.14)	−0.4 (−0.51)		

*h^2^, Communalities; **λ**, Eigenvalue.

**FIGURE 4:**
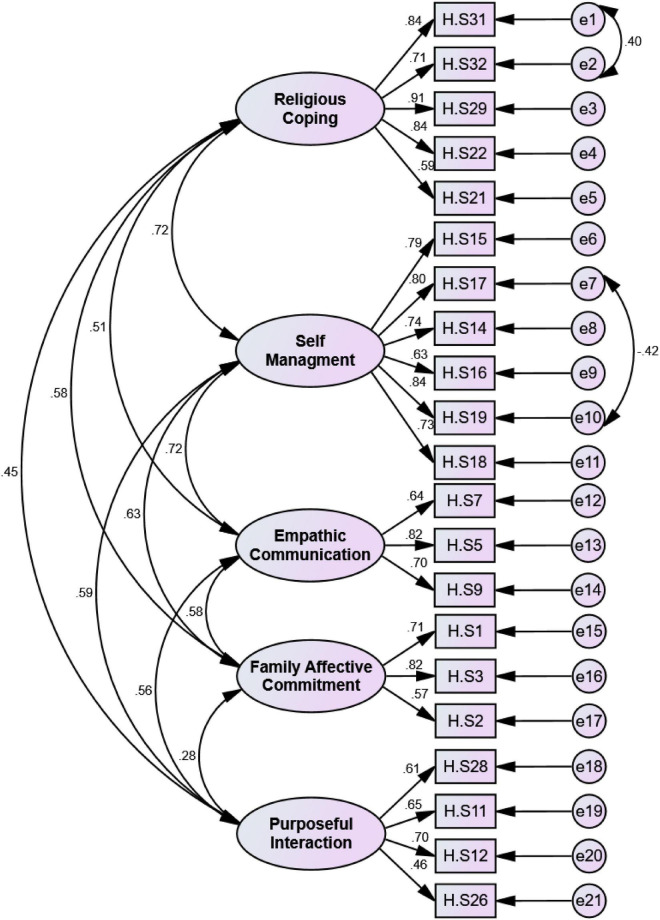
First order CFA of family caregiver hardiness scale (*n* = 225).

In the next step of construct validity, the model was tested by CFA. The results showed all of the model fit indices were in the acceptable range and showed the model of family caregivers’ hardiness is fit (Figure 4). For example, the chi-square model fit index was 311.314 (*p* < 0.001), CMIN/DF was 1.759, RMSEA was 0.065. The results of the other model fit indices are shown in Table 3.

**TABLE 3:** Factors adjustment indexes obtained in exploratory factor analysis of the FCHS (*n* = 225).

	1 Factor	2 Factor	3 Factor	4 Factor	5 Factor (Final model)
CMIN	6.797	90.868	141.204	203.053	311.314
df	3	41	72	111	177
*P* value	0.079	<0.001	<0.001	<0.001	<0.001
CMIN/DF	2.266	2.216	1.961	1.825	1.759
RMSEA	0.083	0.082	0.073	0.068	0.065
PNFI	0.706	0.713	0.720	0.726	0.719
PCFI	0.708	0.715	0.754	0.772	0.784
TLI	0.978	0.946	0.941	0.934	0.916
IFI	0.993	0.960	0.954	0.947	0.931
CFI	0.993	0.960	0.953	0.946	0.930

The first four factors of the scale which had convergent validity based on AVE, MSV, and CR results were used to assess convergent, discriminant validity. All items had discriminant validity. Furthermore, the results of HTMT showed that there are no warnings for discriminant validity (Tables 4, 5).

**TABLE 4:** The indices of the convergent, discriminant validity, and internal consistency of FCHS OF CFA (*N* = 225).

	CR	AVE	MSV	MaxR (H)	Alpha [CI95%]	Omega	AIC
Religious coping	0.889	0.620	0.520	0.917	0.889	0.900	0.615
Self- management	0.890	0.575	0.520	0.898	0.880	0.882	0.557
Empathic communication	0.767	0.525	0.513	0.788	0.764	0.766	0.522
Family affective commitment	0.749	0.504	0.396	0.783	0.749	0.773	0.502
Purposeful interaction	0.699	0.372	0.351	0.716	0.691	0.692	0.364

**TABLE 5:** The results of HTMT of FCHS.

Factors	Religious coping	Self- management	Empathic communication	Family affective commitment	Purposeful interaction
Religious coping					
Self- management	0.755				
Empathic communication	0.526	0.718			
Family affective commitment	0.549	0.643	0.527		
Purposeful interaction	0.429	0.610	0.578	0.296	

The results of Cronbach’s alpha, McDonald’s omega, and AIC for five factors were greater than 0.7 and 0.4, respectively, and the internal consistency of the scale was acceptable. In addition, the scale had a strong coefficient based on the results of CR and Max H reliability (Table 4). Finally, the stability of scale was strong based on the overall ICC result (0.903, 95% CI: 0.719–0.967) (Table 6). Absolute reliability based on SEM results was 2.89. This value indicates that the scale score in a person varies ± 2.89 in repeated tests. Based on the results of MDC, MIC, LOA, and ceiling and floor effect, this scale had responsiveness. In addition, the results of the floor and ceiling effects showed that the items are free of these effects and the scale has interpretability (Table 6).

**TABLE 6:** The results of stability, SEM, responsiveness, and interpretability.

	ICC	SD_pooled_	Mean	SEM	MDC95	MIC	LOA
Scale	0.903	9.31	86.70	2.89	8.01	4.65	68.45 to 104.94

## Discussion

The results of this study indicated that family caregivers’ hardiness concept has five dimensions, namely, commitment, control, challenge, connection, and culture of Iranian caregivers. Therefore, FCHS is a valid and reliable scale for assessing this concept in family caregivers of patients with AD. This scale includes 21 items and five factors, namely, religious coping, self-management, empathic communication, family affective commitment, and purposeful interaction that explained 58.72% of the total variance of this concept. The FCHS model obtained with EFA was confirmed with CFA. As the results of convergent and discriminant validity showed that the factors of this scale correlate with total scale, while they have a low correlation with each other. Therefore, the five factors of this scale are independent.

Since one of the main goals of the factor analysis is to maximize variance, in this study, the variance was 58.72% that factors one and two explained the greatest values of 16.37 and 15.83%, respectively. Among the scales designed to measure the concept of hardiness, regardless of the factor extraction method, two scales explained variance more than FCHS. The Children’s Hardiness Scale (CHS) explained 65.75% (Soheili et al., 2021), and graduate students’ academic hardiness (GSAH) explained 61.87% (Cheng et al., 2019).

Furthermore, this scale had excellent internal consistency based on the results of Cronbach’s alpha, AIC, and McDonald’s omega. It is noteworthy that one of the advantages of this scale is having strong stability based on the value of ICC. Another advantage of this study was the evaluation of measurement error, responsiveness, and interpretation of FCHS. So that the results showed, FCHS has the minimum amount of SEM, responsiveness, and interpretability. SEM indicates the accuracy of the measurement for each individual, and the smaller value of it is important. Responsiveness demonstrates the ability of a scale to show changes in a person’s situation over a period. Finally, the interpretability shows the ability of the scale to show the meaningfulness of changes. These features are an important and required domain of the COnsensus-based Standards for the selection of health Measurement Instruments (COSMIN) CHECKLIST (Terwee et al., 2007) that were not reported in the previous studies of the psychometric properties about hardiness.

The FCHS has five factors, namely, “religious coping,” “self-management,” “empathic communication,” “family affective commitment,” and “purposeful interaction.” The first factor of FCHS was labeled “religious coping.” It includes five items that explained 16.37% of the total variance. The religious coping concept is defined as using religious beliefs or behaviors to facilitate problem-solving to prevent or reduce the negative emotional consequences of stressful living conditions (Koenig et al., 1998). In this scale, religious coping was defined as the caregiver’s ability to use religious and spiritual behaviors and beliefs to cope with the stresses of caring for a patient with AD. It is noteworthy that Mund in 2017 proposed culture as one of the five dimensions of hardiness concept (Mund, 2017); because based on the finding of previous studies, Mund had suggested that a strong background of culture had contributed to the formation of personality and coping strategies. The Iranian culture has been associated with religion and spirituality, and it helps people deal with stressful situations (Abdollahi and Abu Talib, 2015). Our study shows that religion and spirituality had the greatest impact on the hardiness of family caregivers of patients with AD. Therefore, the findings of our study reinforced Mund’s suggestion as an introduction to the fifth component of hardiness.

The second extracted factor was “self-management” with 6 items. In line with the definitions provided for self-management (Barlow et al., 2002), this scale refers to self-management as the psychological mechanisms used to cope with the stresses of caring and to overcome difficult situations including positive thinking, self-remembering and self-emphasis, and patience with the individual to handle their emotions. This factor is related to the control component of hardiness (Kobasa, 1979). Furthermore, the meaning of the self-management factor is in line with the control of affect in the academic hardiness scale (Weigold et al., 2016) studied in GSAH (Cheng et al., 2019), because control of affect also assesses a person’s ability to handle his/her emotions related to academic issues. Since caring for patients with AD has a more psychological burden for caregivers (Fujihara et al., 2019), having the ability to manage this burden is important, and based on the results of this study, self-management was recognized as the second most effective factor.

The third factor extracted was labeled “empathic communication” with 3 items. Empathetic communication is defined as “a two-step process involving: (1) an in-depth understanding of the other person’s problem or feelings; and (2) transmitting this understanding to the individual in a supportive manner and promoting greater satisfaction and acceptance of support in that person” (Pehrson et al., 2016; Kurtz et al., 2017). This scale, based on the content of items 5, 7, and 9, refers to the ability to understand and accept the patient’s problems and to transmit this understanding to the patient in a way that leads to a feeling of satisfaction in the patient. It can be related to the challenge component of hardiness. Empathy or the ability to communicate empathetically with patients with AD is an important part of meaningful care and has been shown to enhance the quality of care and health of the caregiver and patient (Brown et al., 2020).

The fourth factor extracted was labeled as “family affective commitment” with 3 items. Family affective commitment refers to the emotional relationship between family members and being responsible to the family (Tice, 2013). Family caregivers, based on their emotional tendencies and having a sense of responsibility toward the family, engage in the process of caring and maintain the caregiver role despite hardships. Therefore, this factor is related to the commitment component of hardiness.

The final extracted factor was labeled “purposeful interaction” with 4 items. Purposeful interaction, based on its definition in the literature (Mehall, 2021), refers to the caregiver’s ability to connect with physicians, nurses, family members, and friends to gain information and to improve caregiving abilities and reduce the burden of care and situational stress. According to Maddi’s suggestion, the connection can be introduced as the fourth component of the hardiness concept. Maddi believed that interpersonal connection could be an important and influential factor in people’s hardiness in dealing with stressful situations because people gain their strength and ability to deal with stressful situations as a result of connecting with others such as family members and members of society. Based on the items’ content of this factor, family caregivers of patients with AD also strive to develop their ability to cope effectively with the stresses and challenges of care by communicating with others and gaining information.

## Limitations

One of the important limitations was the concern about the generalization of finding because samples were recruited from Iranian populations. Since culture was recognized as the main factor that affects family caregivers’ hardiness, this scale should be tested in other cultures. Therefore, another limitation related to using the online questionnaire for data gathering is that it is not possible to verify the participants’ answers due to the lack of physical contact.

## Study Strength

Nevertheless, this study has several strengths. One of the important strengths is the innovative methodological approach such as Horn’s parallel analysis and exploratory graph analysis for extracting factor structure. Furthermore, this study assessed the important and required domain of COSMIN CHECKLIST, namely, the assessment of SEM, ICC, responsiveness, and interpretability that had not been reported previously about hardiness scales.

## Implication

The phenomenon of aging and age-related problems such as AD is increasing, and caring for these patients is an overwhelming and a stressful task for family caregivers. Therefore, being aware of the level of the hardiness of caregivers and designing an intervention to improve hardiness can prevent negative complications and help improve the quality of care. Therefore, the FCHS with the fewer items, good variance explained, and being exclusive for this group is a useful scale for nurses, therapists, and researchers.

## Conclusion

The finding of this study showed that the FCHS has five dimensions that can be categorized into three components of the Kobasa model including family affective commitment (related to commitment), self-management (related to control), empathic communication (related to challenge), and two new dimensions proposed for this concept including purposeful interaction (related to connection), and religious coping (related to culture). Also, the FCHS scale is a reliable and valid scale with 21 items for assessing the hardiness concept in family caregivers. Based on the results, culture, especially caregivers’ beliefs, their ability to manage themselves with patience and positive thinking, communicating with others to raise awareness, and commitment to the family have the most effect on their hardiness.

## Data Availability Statement

The datasets generated and analyzed in the course of this study are available from the corresponding author on reasonable request.

## Ethics Statement

The studies involving human participants were reviewed and approved by the Iran University of Medical Sciences (IR.IUMS.REC.1398.1229). The patients/participants provided their written informed consent to participate in this study.

## Author Contributions

All authors contributed in all of stages of this study such as design the study, data collection, analyzing the results, writing the manuscripts, and approving the final manuscript.

## Conflict of Interest

The authors declare that the research was conducted in the absence of any commercial or financial relationships that could be construed as a potential conflict of interest.

## Publisher’s Note

All claims expressed in this article are solely those of the authors and do not necessarily represent those of their affiliated organizations, or those of the publisher, the editors and the reviewers. Any product that may be evaluated in this article, or claim that may be made by its manufacturer, is not guaranteed or endorsed by the publisher.
